# Diabetic macular edema: new concepts in patho-physiology and treatment

**DOI:** 10.1186/2045-3701-4-27

**Published:** 2014-05-14

**Authors:** Xinyuan Zhang, Huan Zeng, Shian Bao, Ningli Wang, Mark C Gillies

**Affiliations:** 1 Beijing Institute of Ophthalmology, Beijing Tongren Eye Center, Beijing Tongren Hospital, Capital Medical University; Beijing Ophthalmology & Visual Sciences Key Lab, Beijing 100730 PR China; 2 Macula Research Group, Save Sight Institute, The University of Sydney, Sydney, NSW, Australia; 3 Discipline of Pathology, School of Medical Sciences and Bosch Institute, The University of Sydney, Sydney, NSW 2006, Australia

**Keywords:** Diabetic retinopathy, Microvasculopathy, Neuronal degenerative diseases, Therapeutic strategy, Apoptosis

## Abstract

Diabetic macular edema (DME), a serious eye complication caused primarily by hyperglycemia, is one of the major causes of blindness. DME, which is characterized by cystic retinal thickening or lipid deposition, is prone to relapse after successful treatment. DME is a complex pathological process caused by multiple factors, including breakdown of the inner and outer blood-retinal barriers, oxidative stress, and elevated levels of vascular endothelial growth factor which have been demonstrated in both preclinical and clinical studies. Starling’s law theory explains many of the features of DME. Early detection and treatment of DME can prevent vision loss. Current effective interventions for DME include treatment of systemic risk factors, such as elevated blood glucose, blood pressure and dyslipidemia. Ophthalmic treatments include laser photocoagulation, surgery and intraocular pharmacotherapy. New drugs, which are given by intraocular injection, have emerged in recent years to become first line treatment for DME that affects the central macula with loss of vision. Laser photocoagulation is still the gold standard of treatment for DME which does not involve the central macular. This review outlines these new treatments with particular emphasis on the optimal timing of how they are given.

## Introduction

Diabetic retinopathy (DR) and diabetic macular edema (DME), serious eye conditions caused primarily by hyperglycemia, are the major cause of loss of vision and blindness in the working population of developed countries [1]. The pathogenesis of DME has not been fully elucidated since it is caused by complex pathological process with many contributing factors. Dysfunction of the inner and outer retinal barriers leads to accumulation of sub- and intra-retinal fluid in the inner- and outer-plexiform layers. Vascular endothelial growth factor (VEGF) has generally been accepted as the main factor that disrupts the inner blood-retinal barrier (BRB) function, making it an important target for pharmaceutical intervention [2].

Breakdown of the outer, especially the inner retinal blood barrier is an early event in the pathogenesis of DME [2]. Hypoxia, ischemia, oxygen-free radicals and inflammatory mediators are all involved in the breakdown retinal blood barrier (BRB). Muller cell, pericyte and glial cell dysfunction combined with vitreous changes are involved in the occurrence and development of macular edema. Chronic hyperglycemia, hypertension and high cholesterol are also important factors related to the incidence of macular edema [3].

A large body of clinical data has confirmed that early detection and treatment of DME is an effective strategy to prevent vision loss [4]. Effective systemic interventions for DME include control of blood glucose, blood pressure and dyslipidemia. Ophthalmic treatments are laser photocoagulation, surgery and intraocular pharmacotherapy. Laser photocoagulation is still the gold standard for DME which central macular is not involved. This review outlines new treatment strategies, with particular emphasis on the optimal window for a variety of therapeutic DME interventions.

## Epidemiology of DME

DME is the major cause of vision loss associated with DR. There are approximately 93 million people with DR, 17 million with proliferative DR, 21 million with DME and 28 million with VTDR, the overall prevalence of DME is 6.81% (6.74–6.89) for DME in people with diabetes worldwide [5], accounting for 12% of new cases of blindness annually [6]. According to studies of the natural history of DME, 24% of eyes with DME will lose at least three lines of vision within3 years [7].

The prevalence of DME depends on the type and duration of diabetes. In patients with type I diabetes, DME occurred in the first 5 years following diagnosis of diabetes, with the prevalence gradually increasing to 40% over 30 years. The Diabetes Control and Complications Trial (DCCT) group reported that the incidence of DME in type I diabetes patients with a 9-year diabetic history was 27% [8]. Around 5% of type II diabetes patients had DME when diabetes was diagnosed, gradually increasing to 30% within 25–30 years. A Chinese population-based epidemiological study reported that the prevalence of DME in type II diabetes was 5.89% [9], while it was 4.3% in Beijing metropolitan areas [10] and 5.2% in rural areas [11].

Several systemic risk factors have been identified in population-based epidemiological studies. In patients <30 years old, independent risk factors for DME included duration of diabetes, proteinuria, gender, history of cardiovascular disease, use diuretics and elevated HbA1C. In patients >30 years old, the incidence of DME is associated with longer duration of diabetes, elevated systolic blood pressure and elevated glycosylated hemoglobin. Proteinuria was positively associated in insulin-dependent patients but not in the group that were not using insulin. The prevalence of DME was also significantly associated with high serum cholesterol levels in patients with type I diabetes [12]. A sharp reduction (from 2.3% and 0.9%) in the prevalence of DME was noted in a Wisconsin population with better blood glucose control over two decades, confirming that chronic hyperglycemia is a critical factor in the pathogenesis of DME [13]. According to the new meta analysis data in 2013, All DR prevalence end points increased with diabetes duration, hemoglobin A1c, and blood pressure levels and were higher in people with type 1 compared with type 2 diabetes [5].

## The pathogenesis of diabetic macular edema

Chronically elevated blood glucose, high cholesterol, the accumulation of oxygen free radicals and of advanced glycation end products (AGE)/AGE receptors, protein kinase C (PKC) and other factors have all been implicated in the pathogenesis of DME [3]. These factors ultimately contribute to an increase in VEGF-A expression, resulting in breakdown of the BRB.

### Mechanisms of diabetic blood-retinal barrier breakdown

#### The blood-retinal barrier (BRB)

The concept of the BRB, originating from the discovery of the blood–brain barrier, was first introduced by Ashton in 1965 based on the study of histamine-induced leakage from the ocular vessels [14]. In this study, significant vascular leakage was observed in many compartments of the eye, but retinal vessels were not affected. Shakib and Cunha-Vaz then confirmed the presence of “zonulae occludins” (tight junctions), epithelial cell-like structures between the endothelial cells of the retinal vessels, using electron microscopy [15,16]. The BRB is formed by extensive junctional complexes found between retinal pigment epithelial (RPE) and vascular endothelial cells. These complexes selectively prevent molecules from passing into the extracellular tissue of the retina [17]. The breakdown of BRB results in accumulation of plasma proteins (e.g. albumin) which exert a high oncotic pressure in the neural interstitum, which tends to produce interstitial edema.

#### Tight junctions

Tight junction-associated proteins play a critical role in maintaining the normal biological function of the retina. The tight junctions of the BRB constitute a biological and mechanical barrier to solute flux between cells (*para*-cellular permeability), allowing the organism to control transport of nutrients and waste products through the cell (*trans*-cellular permeability) [18]. Several reviews have outlined the molecular functions of tight junction proteins [17], the signaling cascade from and to the tight junction complex [19] and the modulation of tight junction function in retinal vascular diseases, especially in *in vitro* studies DR [20,21].

**Figure 1:**
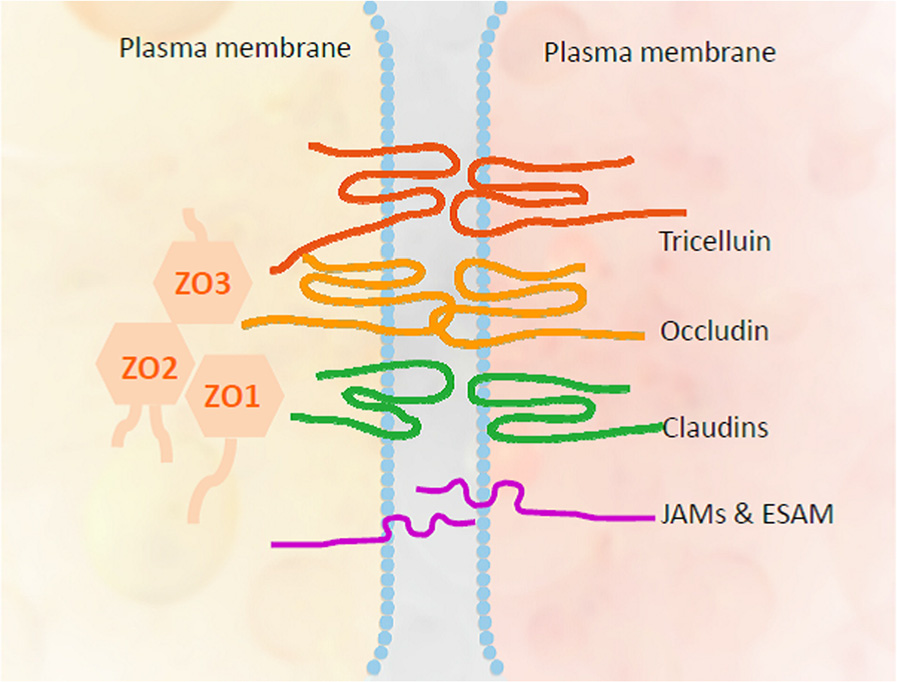
**The inner blood retinal barrier and tight junction proteins.** Three integral proteins form tight junction complexes: occludin, claudins and junctional adhesion molecules (JAMs) & endothelial cell-selective adhesion molecule (ESAM). Tricellulin is a recently discovered tightjunction protein that contributes to the structure and function of tricellular contacts of neighboring cells in many epithelial tissues Occludin and claudins are trans-membrane proteins, predicted to have four trans-membrane and two extra-cellular domains, which are the major structural components of tight junction strands. There are also a group of proteins named membrane-associated guanylate kinase homologs that are positioned on the cytoplasmic surface of junctional contacts. Zonula occludens (ZO) belongs to the MAGUKs family and is thought to interact with occlude.

Three integral proteins form tight junction complexes: occludin, claudins and junctional adhesion molecules (JAMs). Occludin and claudins are trans-membrane proteins, predicted to have four trans-membrane and two extra-cellular domains, which are the major structural components of tight junction strands [17]. Occludin, first discovered as a 65 kDa protein in chicken, has been shown to play an important role in regulating tight junction barrier function [22]. Claudins are a group of proteins that includes 27 members [23]. JAMs belong to the immunoglobulin super-family and are located close to tight junction strands [17,24]. There are also a group of proteins named membrane-associated guanylate kinase homologs (MAGUKs) that are positioned on the cytoplasmic surface of junctional contacts. Zonula occludens (ZO-1) belongs to the MAGUKs family and is thought to interact with occludin [25] (Figure 1).

The functions of tight junction-associated proteins have been investigated *in vitro*. Increased expression of occludin has been shown to correlate with improved BRB function [26]. Functions of claudins may include maintaining the *para*-cellular barrier and regulating *para*-cellular flux through their key selectivity role in the *para*-cellular conductance of ions (such as Na^+^ or Cl^−^) [27,28]. JAMs are thought to interact with other tight junction-associated proteins to regulate barrier function [29]. JAMs have additionally been demonstrated to interact with leukocytes and be involved in mediating inflammatory responses [30,31].

The correlation between tight junction-associated proteins and diabetes-induced breakdown of the BRB has been investigated in several studies. Elevated expression of VEGF-A correlates with increased vascular permeability, concomitant with decreased ZO-1 content in the vitreous of patients with DR [32]. Diabetes also selectively reduces the expression of occludin in the diabetic rat retina in parallel with increased BRB permeability. The consequent increased expression of VEGF-A has also been demonstrated to lead to a rapid phosphorylation of occludin and ZO-1, both *in vivo* and *in vitro*[33]. Expression of ZO-1 and occludin at the cell border is correlated with improved BRB function *in vitro*[34].

In summary, tight junction-associated proteins are key dynamic regulators of the BRB. Dysfunction of these proteins is highly correlated with the pathogenesis of BRB breakdown.

### VEGF-A, a major regulator of blood retinal barrier breakdown in diabetic retinopathy

#### Introduction

VEGF (also referred to as VEGF-A) was first identified as a 34–42 kDa protein in 1983 [35] and cloned in 1989 [36]. On the basis of its ability to induce vascular leakage, measured by^125^I-labeled human serum albumin extravasation, VEGF-A was originally recognized as a ‘vascular permeability factor’ in guinea pigs [35]. On a molar basis, the effect of VEGF-A on vascular permeability is estimated to be 50,000 greater than that of histamine as evaluated by the Miles vessel permeability assay [37].

#### Members of the VEGF family

VEGF-A belongs to the VEGF family that includes placenta growth factor, VEGF-B, VEGF-C, VEGF-D and VEGF-E. Among these VEGF members, VEGF-A has been studied most intensively so far [38,39].

Human VEGF-A comprises at least five different isoforms: VEGF110, VEGF121, VEGF165, VEGF189 and VEGF206.An alternative distal splice acceptor site in exon 8, named VEGF_165b_, which is an inhibitory splice variant of VEGF-A was identified by Bates et al. [40]. The expression of VEGF_165b_ was further evaluated in normal and diabetic human eyes, including the lens, sclera, retina, iris and vitreous. VEGF_165b_ was detected predominantly in normal but not in diabetic vitreous. About 65% of total VEGF-A in normal vitreous is VEGF_165b,_ confirming that VEGF_165b_ is regarded as the endogenous inhibitor of VEGFA [41].

#### VEGF receptors

Three tyrosine kinase receptors have been identified with functionality that corresponds to the VEGF family members. VEGF-A receptor-1 (fms-like tyrosine kinase-1, FLT-1) and VEGF-A receptor −2 (fetal liver kinase-1, FLK-1) are activated by VEGF-A. FLK-1 has been also recognized as a vascular permeability factor, since one of the critical functions of FLK-1 is to regulate vessel permeability [42]. FLT-1 has been reported to be a negative regulator and a ‘decoy’ receptor of FLK-1 by several studies [2,43]. The third receptor, VEGF receptor-3 (fms-like tyrosine kinase-4, FLT-4) is thought to bind to VEGF-C and VEGF-D [44]. Additionally, two co-receptors for VEGF-A, neuropilin-1 (for VEGF165) and −2 (for VEGF145 and 165) have also been identified as the isoform-specific receptors in embryonic vessel formation [45].

#### Role of VEGF-A and its receptors in the pathogenesis of DR and the breakdown of the BRB

It has been well accepted that FLK-1 is the principle mediator of VEGF-A’s effect on vascular permeability and angiogenesis [46]. VEGF-A and FLK-1 have been studied as vascular permeability inducers in different ischemic ocular diseases [2], but the function of the first VEGF-A receptor, FLT-1, is still controversial.

Several hypotheses have been proposed to account for the mechanism by which VEGF-A and its receptors contribute to BRB dysfunction and development of DR, which has also been extensively reviewed [47] and thus will not be the focus of this review.

### Leukostasis and inflammatory cytokines

Leukostasis, the accumulation of leukocytes on the luminal surface of the retinal capillaries, is thought to be a major contributor and early event in BRB dysfunction [48]. Leukocyte adhesion causes endothelial dysfunction and capillary non-perfusion in several ways.

Firstly, it has been demonstrated that leukostasis contributes to DR through the up-regulation of intracellular adhesion molecule (ICAM)-1, a critical molecular player in leukostasis which mediates the adhesion of monocytes and neutrophils to vascular endothelium. ICAM-1 has been found to mediate retinal leukostasis, vascular permeability and BRB breakdown in diabetes. The expression ofICAM-1 is also significantly elevated in STZ-induced diabetic retinas [49] as well as in human diabetic retinas [50]. Furthermore, intravitreal treatment with glucocorticoids has been found to significantly attenuate the inflammatory responses concomitant with improved BRB function through the inhibition of ICAM-1 expression in STZ-rat retinas [51].

Secondly, BRB breakdown resulting from leukostasis may be due to its interaction with VEGF-A. VEGF-A has been shown to up-regulate the expression of adhesive molecules *in vitro*, promoting inflammatory cell adhesion to endothelium [52]. *In vivo*, increased expression of neutrophil CD11a, b, and 18, together with endothelial nitric oxide synthase (eNOS), was induced by VEGF-A in diabetic rat retinas [53]. It has also been shown that the principle pro-inflammatory cytokine, TNF is a mediator of VEGF-A induced BRB breakdown *in vitro*[54]. Elevated expression of ICAM-1 stimulated by VEGF-A was found to be attenuated by pigment epithelium-derived factor (PEDF) in a dose-dependent manner in STZ-diabetic rat retinas [55]. Furthermore, inflammation and BRB dysfunction have been demonstrated to be abrogated by anti-VEGF165 (164) aptamer (EYE001) treatment of diabetic retinas, suggesting that the effect of VEGF-A on leukostasis is highly correlated with the pathogenesis of DR [56].

Thirdly, leukostasis has been found to correlate with inter-endothelial tight junction complex dysfunction and disorganization. Leukostasis was found to induce elevated expression of β-catenin and plakoglobin as well as the disorganization of the vascular endothelial-cadherin/catenin complex, all of which were abrogated by a leukostasis inhibitor (an anti-integrin β monoclonal antibody) *in vivo*[57].

Finally, leukocytes produce reactive oxygen species (ROS) and inflammatory cytokines following binding to the vascular endothelium, leading to increased vascular permeability [58]. There is evidence that the BRB can be preserved by non-steroidal anti-inflammatory drugs (aspirin, etanercept and meloxicam) by preventing retinal vascular leakage through the suppression of TNF [59]. The significance of leukostasis in the pathogenesis of DR provides new insights for the treatment of DR.

Animal experiments confirmed that serum elevated glucose can promote (interleukin-6, IL-6), tumor necrosis factor (TNF), Lymphotoxin and cyclooxygenase-2 (COX-2) expression [8]. The upstream inflammatory cytokines of VEGF-A induce VEGF-A activation, which in turn leads to the destruction of the BRB. It has been found that IL-6, monocyte chemotactic protein-1 (MCP-1), and PEDF expression was significantly increased in the vitreous [17].

### Nitric oxide (NO)

In the late 1980s, Furochgott and Zawadzki (1980) found that vascular endothelial cells produce a substance which may induce vascular smooth muscle relaxation. In 1987, it was confirmed, and named the CM 17 endothelial cell-derived relaxing factor (EDRF) [18]. Subsequently, it was discovered that eNOS is closely related to metabolic abnormalities and cardiovascular diseases and is an important neurotransmitter involved in a variety of cellular responses. eNOS is highly correlated with the retention of leukocytes in the microcirculation and destruction of the BRB [19]. Awata *et al.* also showed that polymorphisms of the eNOS gene are one of the most important factors in the pathogenesis of DME. eNOS gene polymorphisms not only play an important role in the occurrence and development of the DME [20], but are also highly correlated with the breakdown of the BRB. BRB breakdown is also accompanied by the up-regulation of ICAM-1 and decreased expression of tight cell junction protein ZO-1. In diabetic animals, vascular leakage was significantly reduced and the BRB was protected by the NOS inhibitor L-NAME [21], verifying the biological roles for eNOS in the pathogenesis of DME, including: (1) induction and retention of inflammatory cells in the microcirculation of the eye; (2) a direct effect on cell junction proteins, decreasing the expression of cell junction proteins; and (3) increasing the expression of VEGF-A which leads to the destruction of the BRB.

### Retinal neurovascular unit

**Figure 2:**
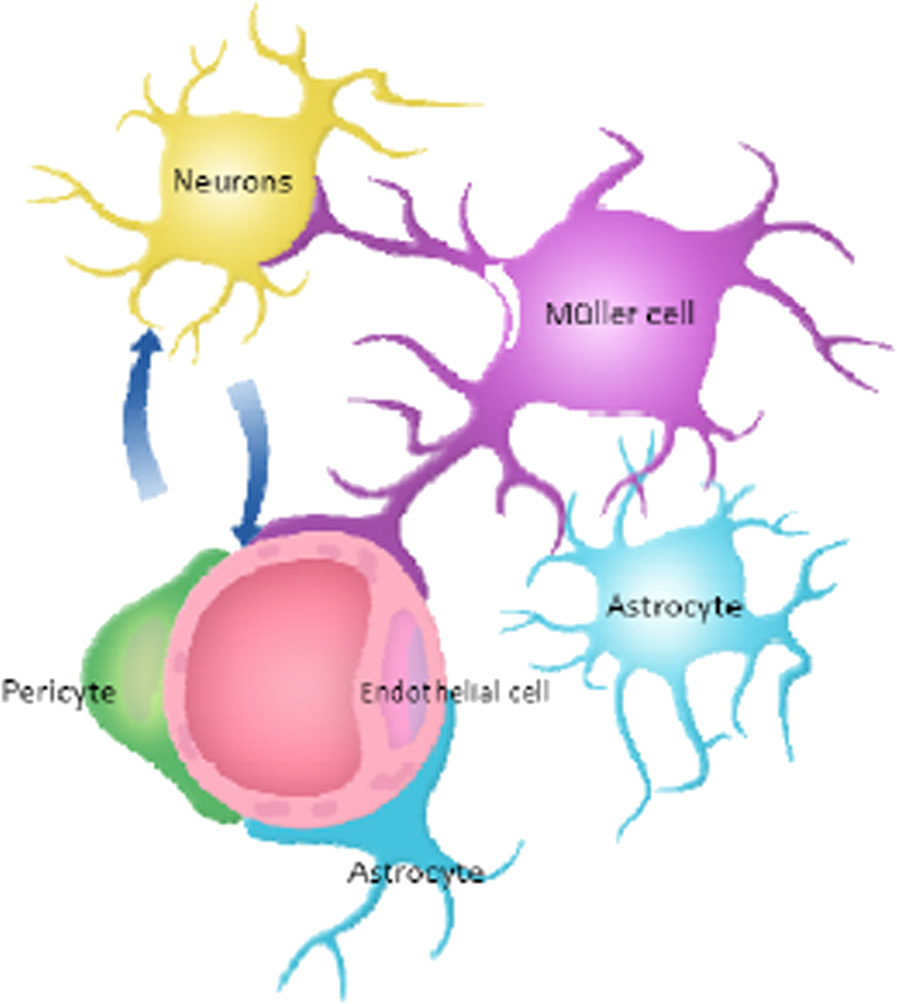
**Retinal neurovascular unit.** The retinal neurovascular unit contains the retinal blood retinal barrier, pericyte, glial cells (astrocytes and Muller cells), neurons.

The nervous and the vascular systems are two parallel systems during embryonic development. They mutually support each other to achieve the promotion of the formation of blood vessels and nerves occur. In relating the physiological functions, retinal neurons rely on their close link to ensure the supply of oxygen and nutrients from the micro-vascular; vasodilation of the blood vessels rely on physiological activities of the nerves; anatomically, retinal neurons, pericytes, mullers, micro-vascular as well as astrocytes are close located. In our previous study, it has been found that neuronal apoptosis and micro-vascular leakage are mutual coexist and interactions. In addition, our study also revealed that VEGFA is a micro-vascular leakage and neuronal apoptosis inducer, both micro-vascular and neurons are effected by VEGFA. The concept of neurovascular unit which contains the brain blood retinal barrier, pericyte, atrocyte, neurons, was originally from the study of stroke, the retina is an extension of the brain and is a part in the visual pathway; furthermore, there are many similarities between the microstructure of brain and retina, such as existing of the blood-retinal barrier, glial cells, pericytes surrounding neurons, and other anatomical structures, based on the above points, we proposed a concept of “retinal neurovascular unit” as long with Antonitte’s group [60] (Figure 2).

Among the retinal neurovascular unit, Muller cells (glial cells) are located between the retinal neurons, functioning as an exchange bridge in the microcirculation and between neuronal cells. Muller cells are also considered to be an important component of the BRB, as anatomically, Muller cell synapses surround the inner retinal blood vessel walls. Cytoplasmic swelling of Muller cells is an early histopathological change in macular edema, resulting in the intracellular accumulation of extracellular fluid. It has been found that the expression of cell junction protein ZO-1 is increased, the normal BRB function is maintained by Muller cells. ZO-1 and occludin have been recognized as important tight junction proteins; the interactions between ZO-1, occludin, as well as perivascular Mullers, glial cells and pericytes play a very important role in maintaining the normal function of the BRB [22]. In addition, under pathological conditions, several growth factors are secreted from the perivascular astrocytes including basic fibroblast growth factor (bFGF), interleukin-1 (IL-1), transforming growth factor-β (TGF-β), vascular endothelial growth factor (VEGF), r-interferon (IFN-r), TNF and insulin-like growth factor (IGF-1), resulting in the breakdown of the BRB [23-26].

### Protein kinase C

More details can be found in Section "Hyperglycemia and its metabolic pathways 1".

### Hyperglycemia and its metabolic pathways

**Figure 3:**
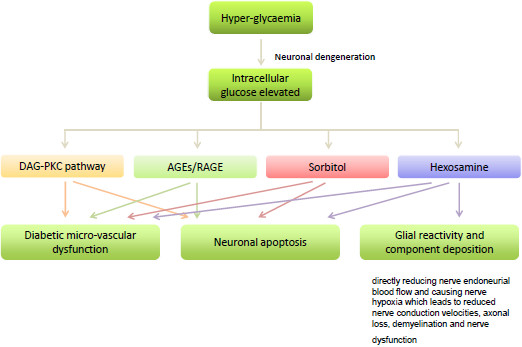
**Hyperglycemia and its metabolic pathways.** Four distinct glucose metabolic pathways are activated by hyperglycemia: Diacylglycerol (DAG)–protein kinase C (PKC), advanced glycation endproducts/ receptor for advanced glycation end-products, Polyol (sorbitol) and Hexosamine pathways. Activation of the metabolic pathways leads microvascular dysfunction, neuronal apoptosis, glial reactivity and component deposition.

Whilst the etiology of DR is highly complex and not fully understood, hyperglycemia has been accepted as the major pathological factor contributing to the development of DR. Four distinct glucose metabolic pathways are activated by hyperglycemia (Figure 3):

1 Diacylglycerol (DAG)–protein kinase C (PKC) pathway. Hyperglycemia increases synthesis of DAG via the *de novo* pathway, which in turn activates PKC isoforms reviewed [61]. The pathogenic role of the DAG-PKC pathway in the pathogenesis of DR has been demonstrated in both human and animal studies [62,63].

PKC, one of a family of serine/threonine protein kinases of which there are at least 12 known isoforms, has been implicated in the pathogenesis of diabetic BRB breakdown both *in vivo* and *in vitro* through a variety of mechanisms [64]. Firstly, its effect is mediated via VEGF-A [65]. The regulation of VEGF-A gene expression has been shown to be controlled and enhanced by PKC-β in a transgenic mouse model [66]. The mitogenic effects of VEGF-A are also mediated by the activation of PKC-β *in vitro*. Secondly, PKC can be activated by oxidative stress through ROS produced by hyperglycemia or advanced glycation end-products (AGEs), shown to directly activate PKC [67]. Thirdly, PKC triggers phosphorylation of tight junction-associated proteins to induce BRB breakdown. Phosphorylation of occludin and ZO-1 was shown to correlate with the activation of PKC in diabetic BRB dysfunction in STZ rats [68]. On the other hand, increased vascular permeability was shown to be suppressed by a PKC-β selective inhibitor, ruboxistaurin mesylate (LY333531) in diabetic rat retinas [69]. The oral administration of this inhibitor has been studied in clinical trials of DR. An initial report from this phase III clinical trial suggested that administration of 32 mg LY333531 per day may reduce vision loss in patients with DME, however, this trial did not meet its primary endpoint (The PKC-DRS Study Group, 2005). Another phase III randomized clinical trial on the effect of ruboxistaurin on progression of DME is underway.

PKC is also involved in hyperglycemia-induced retinal neuronal apoptosis. This may be mediated through inhibition of Akt-mediated signaling pathways modulated by the activation of PKC-δ *in vivo*[70]. Phosphorylation/activation of the apoptotic regulator p38MAPK in response to hyperglycemia stimuli was also found to be mediated through activation of PKC [71].

2 Advanced glycation end-products. Intracellular elevated glucose reacts non-enzymatically with the amino group of proteins, lipids and nucleic acids to form a reversible Schiff base, which is subsequently converted to the stable Amadori product (glycation product) and further metabolized to AGEs [72]. AGEs modulate cellular function mediated through binding of their specific acceptor molecules. Receptor for AGE (RAGE) was identified and characterized as a 35 kDa, lactoferrin-like AGE binding receptor expressed on endothelial cells [1].

Binding of AGEs by RAGE leads to endothelial dysfunction and BRB breakdown in DR. In a RAGE transgenic mouse model, AGEs/RAGE interaction was shown to induce leukostasis and BRB breakdown, which was attenuated by a soluble form of RAGE [73]. Dysfunction of endothelial progenitor cells was found to be induced by AGEs/RAGE through the p38MAPK pathway [74]. AGEs/RAGE interaction is also believed to trigger oxidative stress [75], the release of pro-inflammatory cytokines [76] and increased expression of VEGF-A [77], leading to further diabetic BRB breakdown and neuronal degeneration in the retina.

AGEs are neurotoxic to retinal neurons. *In vitro*, retinal neuronal cell death induced by AGEs and hyperglycemia has been shown to occur in a time- and dose-dependent manner and be mediated through the activation of ROS, suggesting oxidative stress is a consequence of AGEs/RAGE interaction [78]. Both AGEs and ROS have been demonstrated to induce retinal ganglion cell degeneration, possibly mediated by PI3 kinase-dependent pathways [75].

3 Polyol (sorbitol) pathway. Hyperglycemia leads to elevated levels of intracellular glucose, which is then converted to sorbitol by the enzyme aldose reductase using nicotinamide adenine dinucleotideüü phosphate as a cofactor. Sorbitol is subsequently metabolized to fructose, a step which is rate-limiting.

Activation of the sorbitol pathway leads to DR. Activation of the enzyme aldose reductase and accumulation of sorbitol was found in retinal capillary pericytes of human diabetic and STZ-rat retinas [79,80]. Excess accumulation of sorbitol and fructose have been demonstrated to correlate strongly with diabetic micro-vascular dysfunction [81], neuronal apoptosis [82], glial reactivity and complement deposition [83]. The selective aldose reductase inhibitors fidarestat and aldose reductase inhibitor-809, have been demonstrated to significantly abrogate neuronal apoptosis by inhibition of oxidative-nitrosative stress and glial cell activation in STZ-induced diabetic rat retinas [83,84].

4 Hexosamine pathway. Hyperglycemia induces mitochondrial superoxide over-expression, and leads to the activation of the hexosamine pathway [67]. Activation of this pathway has been found to induce oxidative stress [85], production of some pro-inflammatory cytokines such as TGF-α [67], −β [86] and plasminogen activator inhibitor [87], which subsequently induce diabetic retinal neuronal apoptosis [88], endothelial dysfunction [89] and BRB breakdown [90].

In peripheral nerves, hyperglycemia is associated with neuronal degeneration by directly reducing nerve endoneurial blood flow and causing nerve hypoxia which leads to reduced nerve conduction velocities, axonal loss, demyelination and nerve dysfunction [91,92].

### Hydrodynamic principles

#### The starling principle

Capillary fluid movement is composed of three processes: diffusion, filtration and pinocytosis. Starling’s equation reflects the mechanism of fluid filtration across the vascular membranes. The Starling principle emphasizes the pressure difference between the hydrostatic and osmotic forces of liquid flow as the driving force which contributes to macular edema. The Starling equation is as follows:

$$\text{Jv}=K_{f}\left(\left[P_{c}-P_{i}\right]-\sigma \left[\pi_{c}-\pi_{i}\right]\right)$$

Where *J*_*v*_ is the net fluid movement between compartments, [*P*_c_–*P*_i_]–*σ* [π_c_–π_i_] is the net driving force, *P*_c_ is the capillary hydrostatic pressure, *P*_i_ is the interstitial hydrostatic pressure, π_c_ is the capillary oncotic pressure, π_i_ is the interstitial oncotic pressure, *K*_f_ is the filtration coefficient – a proportionality constant, and *σ* is the reflection coefficient.

Intravascular fluid penetrates into the tissue, results in liquid accumulation in the outer plexiform layer of the macular area caused by the high intravascular osmotic pressure due to high blood glucose. Retinal arterioles also contribute to increased vascular resistance and maintain the downstream pressure balance, causing dilation of small caliber arteries. According to the Starling principle, along with the pressure decrease of the branch of the ciliary vessels, the resistance decreases, the consequence of which induces fluid retention between cells.

#### The Young-Laplace equation

The Young-Laplace equation, a nonlinear partial differential equation also describes the papillary pressure difference over an interface in fluid mechanics. The Young-Laplace emphasizes aspect of static capillary surface and elucidate the pressure difference is due to the shape of the surface or wall. The equation is described as follows:

$$\begin{array}{ll} \;\Delta p & =-\gamma \nabla \cdot \hat{n}\\ & =2\gamma H\\ & =\gamma \left(\frac{1}{R_{1}}+\frac{1}{R_{2}}\right) \end{array}$$

Under the condition that only normal stress( in the absence of tangential stress) is considered, Δp is the pressure difference across the fluid interface, y stands for the wall tension, $\hat{n}$ is the unit normal pointing out of the surface, *H* is the mean curvature, and *R*_1_ and *R*_2_ are the principal radii of curvature.

### Vitreous

Vitreous is located just behind the lens and in front of the retina, acting as a buffer to resist external forces and seismic effects. Containing viscoelastic resin, soluble proteins, glucose, free amino acids, and electrolytes, vitreous is a major component of the refractive substance in the eye. Due to the important anatomical correlation with the retina, it also functionsin metabolism and transportation – the macromolecules in the retinal vascular are prevented from moving into the vitreous by the BRB, while normal vitreous can inhibit the proliferation of a variety of cells to maintain intravitreal environmental stability. A large number of studies have confirmed that the vitreous plays an important role in the pathogenesis of DME. In Ouchi*etal*’s study using two-dimensional gel electrophoresis and mass spectrometry, eight proteins in the DME group were significantly up-regulated, including six major cytokines of PEDF, Apolipoprotein A-IV (APoA-4), ApoA-1, Trip-11, and plasma retinol-binding protein, suggesting that these proteins are involved in the pathogenesis of DME in patients with DR [93]. It was also found that the expression of PEDF in DME vitreous was significantly increased, suggesting that vitreous inflammation plays an important role in the pathogenesis of DME [50,93].

The interaction between the vitreous and retina has been found to be involved in the development of macular edema; in particular, when the vitreous and the macular area of the retina are tightly conjugated, macular edema can be greatly promoted [94]. Posterior vitreous detachment occurs when the posterior cortex of the vitreous separates from the internal limiting membrane for several reasons. It was found that the incidence of DME is much lower in diabetic patients with PVD compared with those subjects without PVD (20% versus 55%), suggesting a strong protective effect of PVD [95] This conclusion has also been confirmed by Okulistycznego and his colleagues [96].

In summary, the proposed mechanism of vitreous in the pathogenesis of DME may be due to: (1). the mechanical tractional effects of the epiretinal membrane (ERM). Breakdown of the BRB results in the accumulation of a large number of inflammatory cytokines in the posterior vitreous cortex and is highly correlated with ERM formation. Tractional effects of the ERM in the tangential direction of the macular area aggravates existing macular edema.(2) The direct effects of cytokines due to the breakdown of the blood-vitreous barrier. Breakdown of the vitreous-retinal barrier is correlated and is the sequence of breakdown of BRB. Accumulation of growth and inflammatory factors in the macular posterior vitreous cortex contributes to the formation of EPM, eventually aggravating macular edema.

### Genetic factors

It is well accepted that susceptibility to DME is attributed to a combination of both genetic and environmental factors in individuals.

DR and DME are caused by complex genetic and environmental factors, varying between individuals. Homozygosity of the methyl-enetetrahydrofolatereductase gene (677 T/677 T) polymorphism was shown to correlate highly with the progression of DR in patients with type II diabetes, especially in those with poorly controlled serum glucose [97]. Polymorphisms of eNOS [98], obesity-related genes (db/db and ob/ob) [99], TGF-β1 and -β2 stimulated clone-22 genes [100], have also been proposed to contribute to the development of D polymorphisms in erythropoietin [34], eNOS [20] and VEGF-A genes, and are considered important in the pathogenesis of DME. A meta-analysis of population-based studies (973 patients, 1856 controls, SNP2543887) has shown that the rs476141 gene which is located in chromosome 1, is closely related to the pathogenesis of proliferate DR and macular edema (sight-threatening retinopathy) [35]. VEGF-B gene also promotes BRB breakdown and the formation of retinal neovascularization [36].

## Diagnosis and classification of diabetic macular edema

DME is clinically classified as diffuse, focal or both. DME is characterized by microaneurysm formation and diffuse leakage from the retinal capillaries or even arterioles [101]. Diffuse leakage may also come across the RPE due to the dysfunction of RPE transport induced by degeneration of RPE cells and choroidal vascular insufficiency [102]. In this type of DME, cystoid macular cavity formation can be detected by OCT and/or FFA [103]. Focal macular edema is characterized by the presence of microaneurysms and hard exudates rings or ‘circinate exudates’, surrounding the leaking microaneurysms [103].

Four categories of DME have been established based on OCT: (1) diffuse DME (DRT) which is characterized by retinal thickening, weakening of the light reflection and irregular low reflex zone; (2) cystoid macular edema (CME): a cystoid dark cavity can be visualized; (3) serous sensory detachment DME (SRD) which is characterized by neuronal sensory or even pigment epithelial detachment which can be visualized by OCT; (4) vitreomacular traction(VMIA): this type is characterized by incomplete or complete posterior vitreous detachment, and ERM formation or vitreomacular traction or both exists [37].

### Clinically significant macular edema (CSME)

The *Early Treatment Diabetic Retinopathy Study Group* defined the criteria for ‘clinically significant macular edema’ as having any of the following characteristics [104]:

1. Thickening of the retina at or within 500 microns of the center of the macula

2. Hard exudates at or within 500 microns of the center of the macula, if associated with thickening of adjacent retina (excluding residual hard exudates remaining after disappearance of retinal thickening).

3. Retinal thickening at one disc area or larger, at any part of which is within one disc diameter of the center of the macula.

## Treatment strategies for diabetic macular edema

**Figure 4:**
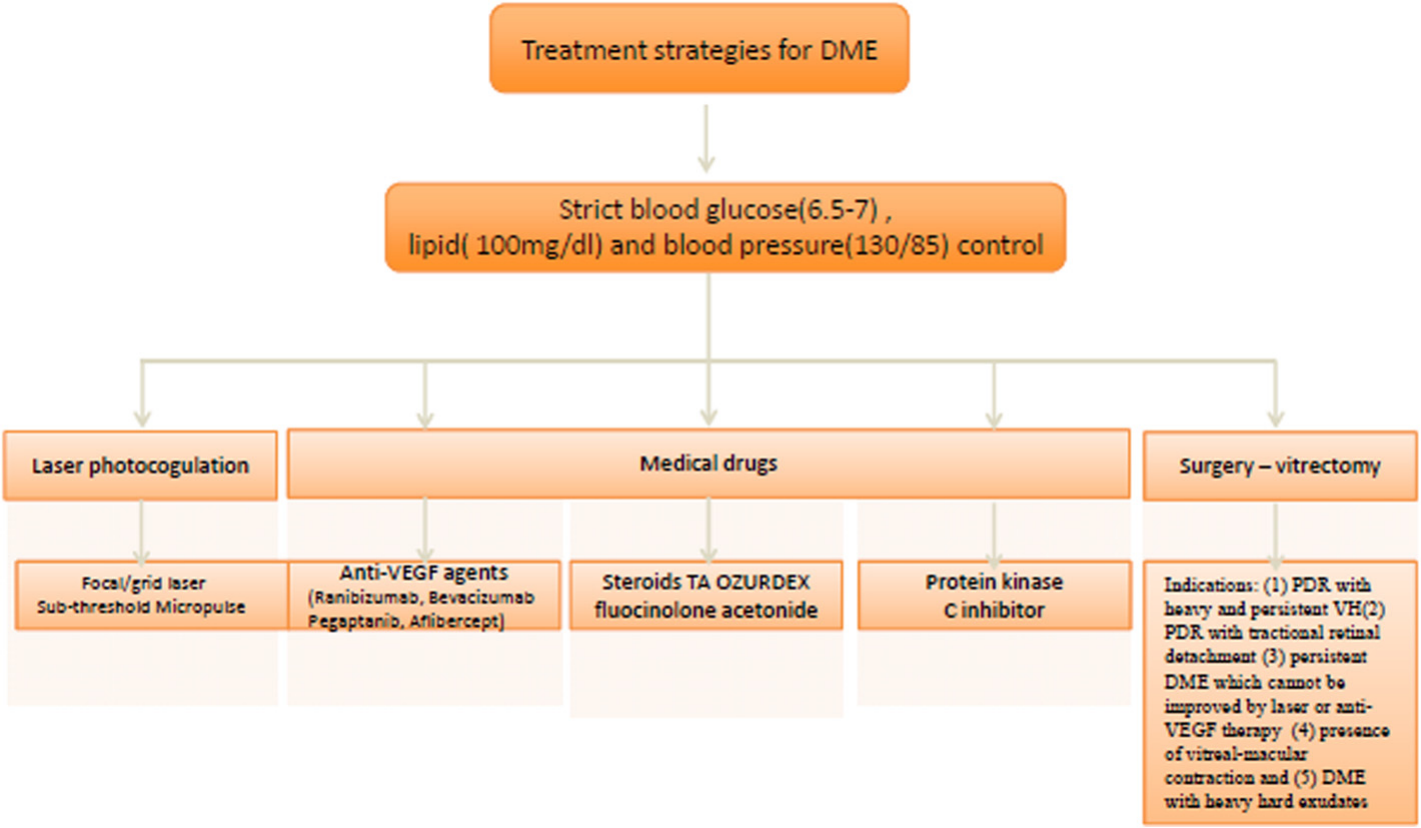
**Treatment strategies for diabetic macular edema.** Strict blood glucose, lipid and blood pressure control is critical for prevention and treatment of DME. According to the recommendations of the American Diabetes Association, HbA1C should be controlled at 6.5-7% and blood pressure should be below 130/85 mmHg, with total lipids lower than 100 mg/dL. Local treatments for eyes with DME include laser photocoagulation, vitrectomy surgery, and intravitreal injection of drugs (glucocorticoid hormone, anti-VEGF agents and PKC inhibitors).

Strict blood glucose, lipid and blood pressure control is critical for prevention and treatment of DME. According to the recommendations of the American Diabetes Association, HbA1C should be controlled at 6.5-7% and blood pressure should be below 130/85 mmHg, with total lipids lower than 100 mg/dL [39]. The purpose of local eye treatment is to reduce swelling, control the progression of the disease, and improve vision. Local treatments for eyes with DME include laser photocoagulation, vitrectomy surgery, and intravitreal injection of drugs (glucocorticoid hormone, anti-VEGF agents and PKC inhibitors) (Figure 4).

### Traditional therapy

Laser photocoagulation remains the gold standard treatment for DME. Focal macular laser treatment was demonstrated to reduce the risk of moderate visual loss in eyes with clinically significant DME from 28% to 11% at 36 months follow-up, in a prospective large-scale randomized clinical trial in the USA [104]. The clinical outcomes of scatter and focal laser treatment were compared in diabetic patients with clinically significant DME. Compared to scatter laser treatment, focal photocoagulation was found to reduce moderate visual loss with no deleterious effects on visual fields over a 5-year follow-up [105]. Newly published data (2008) from the *Diabetic Retinopathy Clinical Research Network* demonstrated that over a 2-year follow-up period, focal/grid laser treatment was more beneficial for visual acuity in patients with DME compared to 1 or 4 mg intravitreal triamcinolone acetonide (IVTA) treatment. In this multi-center clinical trial, 880 eyes of 693 patients with DME were enrolled; OCT was used to monitor the retinal thickness, which was found to parallel the visual acuity results. A significant reduction of retinal thickness from baseline to 2 years was noted in the laser treatment group(139 ± 148 μm), compared with 1 mg (86 ± 167 μm, p < 0.001) and 4 mg (77 ± 160 μm, p < 0.001) IVTA groups, suggesting that laser still remains the benchmark treatment for patients with DME [106]. Pan retinal photocoagulation (PRP) is an effective treatment for both high-risk non-proliferative diabetic retinopathy (NPDR) and proliferative diabetic retinopathy (PDR) to prevent or reverse pre-retinal neovascularization. Focal or grid laser is applied for focal or diffuse macular edema. It has been shown that the risk of vision loss from advanced DR can be reduced by 50% with PRP treatment [107].

Complications associated with photocoagulation in the macula include choroidal neovascularization, hemorrhage, impairment of visual acuity, visual fields and contrast sensitivity [108]. The mechanism of traditional photocoagulation is to reduce the oxygen consumption by destroying the outer segment of photoreceptors, and as a consequence [109], the inner neuronal fiber layer may be damaged during the process of laser penetration. Many efforts have been made to reduce the damage to the retina by modifying the laser parameters, e.g. reduce the exposure timing, use yellow beam, etc. A sub-threshold micro-pulse laser has emerged in the clinic in recent years [110]. This works by reducing the duration of laser exposure using a sub-visible clinical endpoint. The selective damage to the RPE cells may lead to an improved balance in angiogenic factors and cytokine release [111]. The exposure timing includes several on and off phases using a micropulse mode, since the inner retinal temperature must remain sub-threshold to maintain its transparency [112].

### Drugs

#### Anti-Vascular endothelial growth factor (VEGF) and its receptor

In February 2004, Genentech VEGF inhibitor bevacizumab (Avastin, 149 kDa) was fast-tracked for approval by the US FDA. It was approved for joint 5FU-based regimens in the first-line treatment of metastatic colorectal cancer and has become the first approved drug listed for the inhibition of tumor angiogenesis [41]. Another Genentech product, Ranibizumab (Luncentis, 50 kDa), received FDA approval in 2006 for the treatment of wet age-related macular degeneration (wAMD). Anti-VEGF treatment has been confirmed by many clinical trials for the treatment of a variety of retinal diseases, including DME [42], age-related macular degeneration (AMD) [43], and macular edema caused by central retinal vein occlusion [44]. Anti-VEGF antibody is also combined with photodynamic therapy for AMD [45]. However, all the clinical data available so far have been derived from small-scale clinical trials, and further validation for the treatment of vascular disease is needed, including larger sample sizes or multi-center clinical trials [41] (Table 1).

**Table 1:** Current anti-VEGF agents on retinal diseases

**Anti-VEGF agents**	**Mode of action**	**Molecular weight (kDa)**	**The phase III trail**	**Year approved by FDA**
Pegaptanib	28-base ribonucleic aptamer	50	Vision trial	2004 for wet AMD
Ranibizumab (Lucentis, Genetech)	a monoclonal antibody fragment (Fab) derived from bevacizumab	50	Anchor, marina, pier (wet AMD) Ride/rice(DME)	2006 approved by FDA for wet AMD 2012approved by FDA for diabetic macular edema
Bevacizumab (Avastin, Genetech)	Humanized anti-VEGF mAb, specific against VEGF165. It was first used as an adjunct treatment for metastatic colon cancer	149	None for eye diseases	2004 approved by FDA for colorectal cancer
Aflibercept (Elya, (Regeneron Pharmaceuticals) VEGF-Trap	Recombinant VEGFR fusion protein that binds VEGFA and B,PGF	115	VIEW 1 and VIEW2 for wet AMD	2011 approved by FDA for wet AMD 2012 European Medicines Agency approved for AMD
KH902 (Chengdu Kanghong Biotechnology Co. Ltd)	A humanized fusion protein that binds all forms of VEGFA,VEGF receptor 1 and 2 and the Fc portion of IgG1	143	Lamp, phoenix	2013 approved by cFDA for wet AMD

*AMD* age related macular degeneration.

*FDA* food and drug administration.

Pegaptanib sodium (Macugen; Eyetech, USA) was approved by the US FDA in December 2004 for the treatment of wAMD. Pegaptanib is a synthetic anti-VEGF-A aptamer (*mw*50kDa) and is highly selective for VEGF165 subtypes. In phase II and phase III clinical trials it was shown that pegaptanib significantly inhibits new blood vessel formation and improves visual function for patients with wAMD. In a multicenter, randomized, 1-year clinical trial, 36.8% of the eyes with DME experienced improvement of visual acuity to 10 letters (19.7% in the control group) by intravitreal injection of 0.3 mg pegaptanib. This result suggests that pegaptanib is effective in the treatment of DME [46].

In 2011, a new generation of anti-angiogenesis drug developed by the Regeneron biopharmaceutical company (Regeneron Pharmaceuticals, Inc, NY USA) called aflibercept (VEGF-trap), was approved by the US FDA. VEGF trap-eye is a fusion protein, with a molecular weight of115kDa and has high affinity to specific receptor VEGFR1 and VEGFR2 antagonists. Aflibercept has been proven to inhibit a tumor’s progression effectively in the VELOUR phase III clinical trial. Aflibercept combined with 5-FU as the second-line regimen in the treatment of metastatic colon cancer significantly prolonged the patient survival. Intravitreal injection of aflibercept has been approved for the treatment of wAMD with visual acuity significantly improved. Phase III clinical trials are currently being validated for its efficacy in retinal vein occlusion-induced macular edema and DME [47].

#### Glucocorticoid therapy

Systemic or ocular glucocorticoid treatment of eye diseases, including DME has been in use clinically for more than 50 years [48]. The anti-inflammatory [49] and directly anti-VEGF-A [1] effects for protection of the BRB have been confirmed in animal and human studies. Triamcinolone acetonide (TA) is a long-acting non-soluble hormone used since the 1960s for the treatment of a variety of eye inflammatory diseases [50,51]. Sub-Tenon’s injection of TA was also used in the 1990s for the treatment of macular edema secondary to various diseases [52]. It has been shown that intravitreal injection of TA inhibits macular edema [48] and wAMD in a well-designed clinical trial in 2003 [53]. In a 2-year, randomized clinical trial, 56% of eyes had visual acuity improved by 5 lines in the Snellen eye chart, corresponding to a decreased retinal thickness using OCT [48]. It has also been found in several clinical trials that intravitreal injection, rather than other delivery methods, is most effective, with the concentration in the vitreous cavity more than six times higher than that with peribulbar administration (1.22 ± 0.24 mug/mL vs. 0.20 ± 0.11 mug/mL).

Three intravitreal TA injection doses of 1, 4 and 20 mg have been used in clinical practice, with 4and 20 mg considered the most effective treatment doses [54,55]. Four milligrams is the most widely accepted dose reported. Increased intraocular pressure and steroid-induced cataract are the main complications of intravitreal injection with TA. Gillies reported that after 2 years follow-up, a 4 mg intravitreal administration was associated with increased intraocular pressure in 44% of eyes, which required medication to control the intraocular pressure, while 55% of eyes needed cataract surgery [48]. It was also reported that after 20 mg IVTA treatment, the percentage of increased intraocular pressure was 41.2% (>21 mmHg), 11.4% (30 mmHg), 5.5% (35 mmHg), and 1.8% (>40 mmHg). Although the reported incidence of intraocular pressure after IVTA treatment is different, only 1% of the eyes needed trabecular filtration surgery to control the increased intraocular pressure [56].

The lLUVIEN sustained-release fluocinolone acetonide (FA) implant device - a new local via sub-Tenon’s or intravitreal injection drug, has been shown to be effective in the treatment of refractory DME and uveitis. In a 2-year phase III clinical trial, 28.7% of eyes with DME had BCVA improved to 15 letters on the ETDRS visual acuity chart. The major adverse effect related to the implant, as demonstrated in clinical trials, is increased intraocular pressure. Interestingly, it has been shown that intravitreal administration of FA significantly protects retinal neurons in the outer nuclear layer as evidenced by significantly improve db-wave amplitude in electroretinogram (ERG), suggesting its neuronal-protective effect [57].

OZUDEX is a dexamethasone sustained-release biodegradable drug. Intraocular OZUDEX has been shown to be long-acting and effective for the treatment of persistent refractory macular edema caused by branch and central vein occlusion, DME, as well as non-infectious uveitis in phase III clinical trials [58].

### Protein kinase C inhibitor

The PKC inhibitor ruboxistaurin (LY333531) is a PKC-beta specific inhibitor. LY33351 significantly inhibited diabetic BRB breakdown and retinal neuropathy in a pre-clinical animal study [60]. In a 3-year clinical trial to test the efficacy and safety of ruboxistaurin, in 1392 eyes, 10.2% experienced moderate vision loss in the control group; 6.1% experienced up to 15 letters in the study group, 7.4% 11.7%. 26.7% respectively in the control group and 35.6% of the eyes needs to be combined with laser photocoagulation to prevent further loss of vision. The results show that ruboxistaurin combined with laser photocoagulation can effectively prevent the occurrence of DME. However, the results need to be further validated by large-scale multi-center clinical trials.

### Surgery - vitrectomy

Pars plana vitrectomy (PPV) surgery was introduced by Machemer *et al*. in the early 1970s [113,114] and has been widely used ever since. It has been confirmed by several clinical trials that it effectively reduces macular edema, especially CME. The mechanisms by which PPV improves macular edema include:(1) alleviation of the ischemic state of the ischemic area of the retina, and prohibition of the secretion of macromolecules for persistent macular edema; and (2) an increase in the oxygen supply to the retinal surface, enhancing absorption of oxidation of the retina thereby reducing macular edema. In addition, (3) vitrectomy could improve the water solubility of oxygen and other nutrients in the vitreous cavity, thus facilitating the transport of oxygen to the ischemic retinal areas [61].

The indications of PPV for PDR include: (1) PDR with heavy and persistent vitreous hemorrhage; (2) PDR with tractional retinal detachment; and (3) persistent DME which cannot be improved by laser or anti-VEGF therapy [115] (4) presence of vitreal-macular contraction and(5) DME with heavy hard exudates [62].

The internal limiting membrane is a basement membrane between the retina and vitreous, acting as the proliferation of the bracket and participate in the formation of macular degeneration. Dissection of the internal limiting membrane in macular hole surgery is a very important step in treating DME. The effectiveness of PPV surgery for DME has been confirmed by several clinical trials. A study of 26 DME patients who experienced vitrectomy surgery combined with ICGA-stained internal limiting membrane dissection, indicates that vitrectomy combined with internal limiting membrane peeling can reach a better visual acuity prognosis for young diabetic patients with recent vision loss due to macular edema without laser photocoagulation [63]. Evidence from some small-scale clinical trials also indicates that there is no significant difference between the with-or-without limiting membrane peeling groups in the prognosis of visual function [64].

In summary, with the development of molecular biology and basic science, there is now a more in-depth understanding of the pathogenesis of DME. Since BRB breakdown has been recognized as the earliest manifestation of DME, prevention of BRB breakdown has become the target of drug intervention and a hot topic for research. A variety of anti-VEGF-A drugs have been widely used clinically, and some new drugs are in development or in phase I-III clinical trials. Achieving an individual therapy, according to the different complex pathological conditions, has become the priority in the development of an effective drug treatment strategy for DME.

## Abbreviations

AGE: Advanced glycation end products; AMD: Age-related macular degeneration; APoA-4: apolipoprotein A-IV; bFGF: Basic fibroblast growth factor; BRB: Blood-retinal barrier; CCL: Chemokine ligand; COX-2: Cyclooxygenase-2; DAG: Diacylglycerol; DME: Diabetic macular edema; DR: Diabetic retinopathy; EDRF: Endothelium derived relaxing factor; FLK-1: Fetal liver kinase-1; FLT-1: fms-like tyrosine kinase-1; ICAM: Intracellular adhesion molecule; IFN: Interferon; IGF-1: Insulin-like growth factor; IL-1: Interleukin-1; JAMs: Junctional adhesion molecules; MAGUKs: Membrane-associated guanylate kinase homologs; MCP-1: Monocyte chemotactic protein-1; NO: Nitric oxide; OCT: Optical coherence tomography; PEDF: Pigment epithelium-derived factor; PKC: Protein kinase C; SDF-1: Stromal-derived factor-1; TGF-β: Transforming growth factor-β; TNF: Tumor necrosis factor; VCAM: Vascular cell adhesion molecule; VEGF: Vascular endothelial growth factor; wAMD: Wet age-related macular degeneration; ZO-1: Zonula occludens.

## Competing interests

The authors declare that they have no competing interests.

## Authors’ contributions

Dr XZ, HZ, SB, NW, and MG participated in the sequence alignment and drafted the manuscript. All authors read and approved the final manuscript.
